# The training–discovery paradox: Evidence from citizen science

**DOI:** 10.1371/journal.pone.0361148

**Published:** 2026-10-29

**Authors:** Shawn Ogunseye, Jeffrey Parsons, Roman Lukyanenko, Yoones Sekhavat

**Affiliations:** 1 Computer Information Systems, Bentley University, Waltham, Massachusetts, United States of America; 2 Faculty of Business Administration, Memorial University of Newfoundland, St. John’s, Newfoundland, Canada; 3 McIntire School of Commerce, University of Virginia, Charlottesville, Virginia, United States of America; 4 Faculty of Mathematics & Computer Science, Modern College of Business and Science, Muscat, Oman; Education University of Hong Kong, HONG KONG

## Abstract

Training enhances classification accuracy in citizen science by directing visual attention toward predefined features, but may constrain attention to unexpected objects or features. We examined this tradeoff through an analysis of 206 citizen science discoveries and through controlled experiments using eye-tracking and verbal protocols. The analysis of existing discoveries showed that untrained contributors produced a broader range of discovery types, including the majority of opportunistic and serendipitous discoveries. In the primary experimental study, explicitly trained observers showed 34% fewer fixations on non-diagnostic features compared to untrained observers. Training also reduced attention to secondary entities and constrained how observers described novel stimuli, a phenomenon we term classification transference. Trained observers described unfamiliar entities by referencing missing trained-category features rather than reporting new attributes. Implicitly trained observers preserved broader attentional sampling while maintaining sensitivity to variation. The second experimental study provided a robustness check with 84 additional participants using written responses and confirmed the patterns observed in the first experiment. These findings demonstrate that training shapes both where observers look and what they report, establishing a perceptual boundary on discovery potential that conventional accuracy measures cannot detect.

## Introduction

In 2007, a Dutch schoolteacher, Hanny van Arkel, noticed a strange green smudge in a galaxy image while participating in Galaxy Zoo, a citizen science platform designed to support classification of astronomical objects [1]. The object, later named Hanny’s Voorwerp, turned out to be a previously unknown astronomical phenomenon, a glowing cloud of ionized gas illuminated by a quasar that had long since faded [2]. Although clearly visible, the object had not been flagged by other participants, whose attention was shaped by training, which emphasized known galaxy morphologies [3].

Training exists because a knowledgeable observer is expected to produce better data. Van Arkel's case reveals a tension in that expectation. Training increases accuracy in precisely defined tasks yet simultaneously narrows visual attention and reporting patterns in ways that may reduce sensitivity to unexpected phenomena. This tension is particularly acute in citizen science, where explicit instructions and curated examples are used to train participants [4–6]. The narrowing filters out phenomena that fall outside the project's defined scope [7,8], and some of those phenomena are discoveries.

*Discovery*, as we use the term, is the identification of a previously unrecognized fact about the world, whether an entity, pattern, attribute, or relationship. What narrowing puts at risk is the capacity to attend to and report such facts, which we term *discovery potential*. If training systematically constrains it, then the very design approach that ensures data quality may be suppressing the anomalies that produce breakthroughs. Despite the importance of this tradeoff, the perceptual processes that enable discovery have not been examined experimentally in citizen science.

Cognitive science provides a theoretical basis for understanding this tension. Training directs attention, reinforces prior expectations, and tunes perceptual filters toward task-relevant features [9–11]. These cognitive processes shape how observers visually sample their environment and what they subsequently report. What observers expect narrows both what they sample and what they report, drawing attention toward anticipated features and leaving the unexpected unregistered [12]. Training instills such expectations, shaping both where observers look and what they can describe.

This paper provides the first experimental evidence that training in citizen science constrains what observers visually attend to, and through that, what they report, establishing a perceptual boundary on discovery that cannot be overcome by instruction or motivation. We make three contributions. First, we document the patterns of real discoveries in citizen science and show how they vary with training type. Second, we provide experimental evidence that training constrains visual attention itself, using eye-tracking to show that trained observers do not fixate on features that could support discovery. Third, we introduce classification transference, a construct describing how training constrains the language and categories available for expressing novelty. Together these establish a training-discovery paradox.

### Study 1: Discovery patterns in citizen science

We analyzed 206 confirmed discoveries from existing citizen science platforms, classifying each into one of three categories. *Targeted discoveries* directly align with project-defined objectives, for instance, when contributors accurately classify galaxy morphologies using provided criteria. *Serendipitous discoveries* occur when participants identify unexpected but relevant findings within structured tasks [13], such as reporting rare aurora variants in sky-monitoring platforms. *Opportunistic discoveries,* in contrast, emerge entirely outside project expectations, usually from open-ended observation or contributor curiosity, with Hanny's Voorwerp in Galaxy Zoo representing a canonical example. This classification framework enables rigorous assessment of how training modalities influence task performance and the character of discoveries that emerge from different observational approaches.

## Method

To examine how training relates to the kinds of discoveries made in real-world contexts, we analyzed discoveries from eBird, iNaturalist and projects on Zooniverse. These platforms are leaders in citizen science design and are well-documented in both academic literature and public-facing communications [14]. The projects hosted on these platforms differ in how they structure training for contributors. Some emphasize explicit classification rules, others rely on exposure to examples, and some allow contributors to proceed without structured instruction.

Each platform maintains a public record of user-generated discoveries. These include formal acknowledgments in scientific papers, public announcements, and blog posts. We manually reviewed these sources to identify discovery events clearly attributed to contributor observations. This yielded a dataset of 206 distinct discoveries, each linked to its originating project and publicly credited to volunteer contributions. The examples that follow are drawn from this corpus. Hanny's Voorwerp, a massive ionized gas cloud, was identified by a Galaxy Zoo volunteer who noticed an anomalous blue smudge while classifying galaxy morphologies [3]. On eBird, contributors documented the first confirmed breeding record of the Painted Bunting in Nova Scotia, a range expansion not anticipated by the platform's monitoring objectives. Through Planet Hunters TESS on Zooniverse, citizen scientists identified the multi-planet system K2-138 by flagging transit dips in stellar light curves, directly applying the diagnostic criteria they had been trained to use.

Two independent coders reviewed each discovery along with its source materials. For each case, coders determined the type of training provided by the platform and then categorized the discovery. A finding that matched the project's stated objectives was coded as targeted. One that was unexpected but fell within the project's broader scope was coded as serendipitous. One that fell entirely outside the project's scope was coded as opportunistic. Coders were provided with these category definitions and classified each discovery independently, recording a written justification citing specific evidence from the source material. There was a high level of agreement between the coders (Cohen's κ = .987 for discovery type; κ = .981 for discovery basis) and disagreements were resolved through discussion.

Training types were classified as ***explicit*** (structured rules), ***implicit*** (exemplar exposure), or ***untrained*** (no instruction), following established learning taxonomies [15,16]. Explicit training was defined as the provision of structured rules or criteria for identifying entities. Implicit training referred to learning through example exposure without classification criteria. The third category, no training, described discovery contexts in which contributors received neither rules nor guided examples. This dataset enabled us to examine how discovery type varies with the structure of training, across diverse observational domains and platform designs. The full dataset, including discovery names, source platforms, assigned classifications, training types, and coding justifications, is publicly available at https://doi.org/10.5281/zenodo.18647301. All data files and analysis code for both studies are described in S5 Appendix in S1 File.

## Results

Training type was strongly associated with the type of discoveries participants reported. This is consistent with prior work showing that platform-level norms and community expectations can influence both the quantity and nature of ecological observations [17]. Explicitly trained contributors predominantly made targeted discoveries aligned with project goals (132 of 151, 87%), though a minority were serendipitous (15 of 151, 10%). Untrained contributors showed a different pattern, with targeted discoveries accounting for fewer than half of their total (20 of 45, 44%) and serendipitous and opportunistic discoveries making up the remainder. Implicitly trained participants resembled the explicitly trained group, with discoveries that predominantly matched expected outcomes (9 of 10 targeted). A Welch ANOVA confirmed significant differences in discovery outcomes across training conditions, *F*(2, 21.41) = 12.44, *p* < .001, η² = .188. Follow-up Games-Howell comparisons showed significant differences between the explicit and untrained groups (*p* < .001, *d* = 1.17) and between the implicit and untrained groups (*p* = .024, *d* = 0.75), but no difference between explicit and implicit participants (*p* = .819, *d* = 0.11). These results suggest that training is associated with closer adherence to project goals and with a narrower range of discovery types (Table 1).

**Table 1:** Discovery type by training type.

Training Type	Targeted Discovery	Serendipitous Discovery	Opportunistic Discovery	Total
Explicit	132	15	4	**151**
Implicit	9	0	1	10
Untrained	20	15	10	45
**Total**	**161**	30	**15**	**206**

These patterns are potentially sensitive to the distributional structure of the data. Uneven group sizes, particularly the small Implicit group (*n* = 10), raise concerns about statistical stability. To assess robustness, we conducted permutation tests (100,000 permutations) on the overall association and each pairwise comparison, as well as Fisher's exact tests on collapsed 2 × 2 tables (see S1 Appendix in S1 File). The overall association between training type and discovery type was significant (permutation *p* < .001). Pairwise permutation tests confirmed that Explicit and Untrained groups differed significantly (*p* < .001), as did Implicit and Untrained groups (*p* = .029). Explicit and Implicit groups did not differ (*p* = .222). Fisher's exact tests on targeted versus non-targeted discoveries produced consistent results (Explicit vs Untrained: *p* < .001; Implicit vs Untrained: *p* = .013; Explicit vs Implicit: *p* = 1.00). These results support the reliability of all three pairwise comparisons (See Table 2).

**Table 2:** Robustness tests for Study 1 discovery-type comparisons.

Comparison	Permutation *p* (100,000)	Fisher's exact *p* (Targeted vs Non-Targeted)
Overall association	< .001	—
Explicit vs Untrained	< .001	< .001
Implicit vs Untrained	.029	.013
Explicit vs Implicit	.222	1.00

Note. Permutation tests used chi-square as the test statistic on the full 3 × 3 table (pairwise: 2 × 3). Fisher's exact tests collapsed discovery types into targeted vs non-targeted (2 × 2).

Separately from the discovery-type classification above, we examined what feature or entity each discovery was based on. Most discoveries (85%) stemmed from diagnostic attributes of the target object (*n* = 175), while secondary attributes (*n* = 25, 12%) and out-of-scope entities (*n* = 6, 3%) were less common. The K2-138 discovery noted above rests on a diagnostic attribute, the transit dip in stellar light curves that Planet Hunters volunteers are trained to identify. The brown dwarf WISEA 1101 + 5400 was flagged by a Backyard Worlds volunteer who noticed a subtle dipole feature across multiple frames, a secondary attribute not central to the project's main classification task. Hanny's Voorwerp, an ionized gas cloud entirely outside the scope of Galaxy Zoo's galaxy morphology project, represents an out-of-scope entity discovered through open-ended observation. Table 3 shows the distribution of discovery types across platforms.

**Table 3:** Discovery type by platform.

Platform	Targeted	Serendipitous	Opportunistic	Total
Zooniverse	141	15	5	161
eBird	19	12	7	38
iNaturalist	1	3	3	7
**Total**	**161**	**30**	**15**	**206**

Training type and platform are closely aligned in this dataset. Generally, Zooniverse projects employ explicit or implicit training, while eBird and iNaturalist contributors operate with minimal structured instruction. The observed associations between training type and discovery type therefore cannot be separated from platform-level differences in interface design, community norms, and onboarding procedures. Hybrid training approaches across platforms [18,19] introduce further complexity. Yet the patterns are consistent and directional. Across all three platforms, contributors operating without structured training produced proportionally more serendipitous and opportunistic discoveries, while those with structured training produced predominantly targeted findings. This consistency across different domains, interfaces, and contributor populations suggests that training itself may play a role, but observational data alone cannot confirm this. To isolate the effect of training from these platform-level factors, we conducted a controlled experiment in which training type was directly manipulated while task, stimuli, and instructions were held constant. If the patterns observed in Study 1 reflect genuine effects of training and not platform differences, they should replicate under these controlled conditions.

### Study 2: Training effects on perception and reporting

Study 1 classified each discovery by its basis, whether it rested on a diagnostic attribute, a secondary attribute, or an out-of-scope entity. Untrained contributors produced disproportionately more of the discoveries resting on secondary attributes and out-of-scope entities. An attribute and an entity differ in kind. A diagnostic or non-diagnostic attribute is a feature of the object being classified; a secondary or out-of-scope entity is a separate object in the scene. Two established mechanisms could produce that difference, and we introduce a third. *Change blindness* is the failure to detect and report visible variations between sequential visual scenes [20,21]. *Inattentional blindness* is the failure to notice a clearly visible but unexpected stimulus when attention is occupied by a primary task [22,23]. *Classification transference*, which we introduce, is the description of a novel entity in the vocabulary of a trained category.

Discovery requires two things in sequence. An observer must attend to an entity or feature, then describe it in terms that preserve what is novel about it. Change blindness and inattentional blindness govern the first, classification transference the second.

The key terminology we use in our experiment is defined in Table 4.

**Table 4:** Key terminology.

Term	Definition	Example
Diagnostic attributes	Features that define category membership and identify an entity	Blue wings, short tail, light blue body
Non-diagnostic attributes	Visible features of the target object that are irrelevant to category membership	Antenna shape, leg count, body orientation
Attribute variation	A change to an attribute of the target object between the training exemplar and the test image	Body color switched from blue to green
Secondary entities	Objects present in the scene but unrelated to the classification task	A bird in the background sky
Secondary attributes	Features of a secondary entity	The bird's color or posture
Out-of-scope entities	Objects presented for classification that belong to no trained category	The catch items in Fig 2
Fixation count	Number of discrete eye fixations recorded on a given feature or entity	Six fixations on the wing region of one image
Reporting rate	Proportion of features or entities mentioned in participant verbal reports	A feature mentioned on 8 of 13 eligible images gives a rate of 0.62
Classification transference score	Difference between references to missing trained-category features and new attributes reported; higher scores indicate greater reliance on prior training	“It doesn't have blue wings” raises the score; “it has red spots” lowers it

Training directs attention toward diagnostic attributes at the expense of non-diagnostic ones. We therefore expect trained participants to notice and report fewer non-diagnostic variations. For diagnostic variations we expect the opposite, fewer fixations to reach the same reporting accuracy, as trained observers process category-relevant features more efficiently.

***H1: Training and attention to attribute variations (change blindness)***

***H1a*** *: Explicitly trained participants will show fewer fixations on non-diagnostic attribute variations than implicitly trained and untrained participants.*

***H1b:*** *Explicitly trained participants will report fewer non-diagnostic attribute variations than implicitly trained and untrained participants.*

***H1c:*** *Untrained participants will show more fixations on diagnostic attribute variations than explicitly and implicitly trained participants.*

***H1d:*** *Reporting of diagnostic attribute variations will not differ across training conditions.*

Inattentional blindness arises from the selective allocation of limited attentional resources. Prior research shows that stimuli not part of task goals are likely to go undetected, even when salient [24]. In citizen science contexts, training that emphasizes diagnostic attributes may narrow attentional focus, leading observers to deprioritize peripheral or secondary elements that fall outside their trained schemas. This attentional narrowing is expected to produce inattentional blindness, causing trained observers to overlook task-irrelevant but potentially informative entities in a visual scene.

***H2: Training and attention to secondary entities (inattentional blindness)***

***H2a:*** *Untrained participants will fixate more on secondary entities than explicitly trained participants, with implicitly trained participants falling between the two.*

***H2b:*** *Untrained participants will report more secondary entities than explicitly trained participants, with implicitly trained participants falling between the two.*

Classification transference has not, to our knowledge, been previously named or measured, though related constraints on schema-driven reporting have been observed in other domains. Unlike change blindness and inattentional blindness, which constrain what observers perceive, classification transference constrains how they communicate what they perceive.

Several established constructs address related aspects of how prior knowledge shapes cognition. Schema-consistent memory bias describes the tendency to encode and recall information that fits existing frameworks while distorting or omitting information that does not fit these frameworks [25]. Anchoring captures how initial information biases subsequent numerical or evaluative judgments [26]. Verbal overshadowing refers to cases where verbally describing a stimulus impairs its subsequent recognition [27]. Cognitive fluency describes the general ease with which familiar forms are processed [28]. Misclassification occurs when an observer assigns a stimulus to the wrong category [29]. Each of these operates at the level of perception, memory, or judgment.

Classification transference is distinct from the above mechanisms. It operates at the level of expressive output. The observer perceives the novel entity, recognizes that it does not belong to any trained category, yet defaults to the trained framework before ordinary description can occur. In the absence of an alternative framework of equal salience, the observer relies on the only structured one available, the trained schema. The result is that novel entities are described not in terms of what they present but in terms of what they lack relative to trained categories. The trained schema overpowers the observer's natural descriptive capacity.

We predict:

**H3:** Explicitly trained participants will rely most on trained categories when describing novel entities, untrained participants will rely least, and implicitly trained participants will fall between the two.

Together, H1 through H3 test whether training constrains discovery potential at the level of perception (H1, H2) and at the stage of communicating novel observations (H3). Table 5 summarizes the hypotheses and the measure on which each one is tested.

**Table 5:** Summary of Hypotheses.

Cognitive phenomenon	Target feature	Attentional prediction (eye-tracking)	Reporting prediction (verbal protocol)
Change blindness	Non-diagnostic attribute variations	H1a: Explicit < Implicit, Untrained	H1b: Explicit < Implicit, Untrained
Change blindness	Diagnostic attribute variations	H1c: Untrained > Explicit, Implicit	H1d: No difference across conditions
Inattentional blindness	Secondary entities	H2a: Untrained > Implicit > Explicit	H2b: Untrained > Implicit > Explicit
Classification transference	Novel entities (catch items)	Not predicted; the phenomenon operates at expressive output	H3: Explicit > Implicit > Untrained

Note. Attentional predictions are tested with eye-tracking (fixation count and total fixation duration); reporting predictions with the concurrent verbal protocol. The design includes no perceptual measure independent of attention, so the two are tested together.

## Method

Prior to the main experiment, we conducted two pretests to select the observation prompt and set viewing duration (S6 Appendix in S1 File). The prompt “What do you see?” was selected because it elicited the fullest responses across all training conditions, and 40 seconds was established as an appropriate viewing duration, consistent with prior work in citizen science on species identification [30].

We implemented a between-subjects experimental design with three conditions. Participants (*N* = 60) were randomly assigned to Explicit Training, Implicit Training, or Untrained.

In the Explicit Training condition, participants received formal classification criteria defining an artificial insect category named Tyrans. Tyrans were characterized by five specific diagnostic attributes: (1) short tail, (2) light blue body, (3) two or three body buttons, (4) blue wings, and (5) one or two wing rings. Participants reviewed these criteria before beginning the task and clarified their understanding through questions if needed.

In the Implicit Training condition, participants viewed five unlabeled examples of Tyrans without any classification criteria. Participants were encouraged to observe these exemplars closely and infer patterns independently. Only positive exemplars were provided to maintain consistent exposure to diagnostic attributes without introducing extraneous variation.

In the Untrained condition, participants were told only that they would view images and describe what they saw, with no prior instruction or exposure. This condition provided a baseline for measuring the cognitive effects of training.All participants, regardless of training condition, received identical instructions during the observation phase. This ensured that differences in reporting patterns reflect the influence of prior training on visual attention and not variation in task instructions.

### Stimuli and attribute manipulations.

Stimuli consisted of drawings of artificial insects (tyrans) created to eliminate prior domain knowledge as a confound, so that observed differences could be attributed to training condition and not to pre-existing expertise. Stimuli were designed to test three aspects of how training affects observation. First, diagnostic attributes (wing length, wing color, tail shape, body color, body patterns) defined Tyran category membership, while non-diagnostic attributes (antenna shape, leg count, body orientation) were visually prominent but irrelevant for determining category membership. Both were varied between training exemplars and test-phase images to measure whether training affected detection of category-relevant changes and category-irrelevant changes (H1a-d). Second, secondary entities were visually noticeable background objects unrelated to the classification task, included to measure whether training reduced attention to peripheral scene elements (H2a-b). Third, catch items were presented for classification in the position a Tyran would occupy but lacked all diagnostic Tyran attributes, sharing only superficial visual properties such as symmetry. These were used to compute classification transference scores measuring whether training constrained how participants described novel entities (H3).

Each participant viewed twenty distinct images. Sixteen images featured a mixture of Tyran and non-Tyran insects. Thirteen images incorporated controlled modifications in diagnostic and non-diagnostic attributes to test susceptibility to change blindness. Changes included variations such as switching body color from blue to green or modifying antenna shape (Fig 1). Catch items appeared at evenly spaced positions 5, 10, 15 and 20. Because training was manipulated between subjects and every condition saw the identical sequence, presentation order is held constant across groups and cannot contribute to the group contrasts. The regular spacing of catch items distributes the novel-entity probes evenly through the session, so classification transference is measured at comparable points in every participant's sequence.

**Fig 1:**
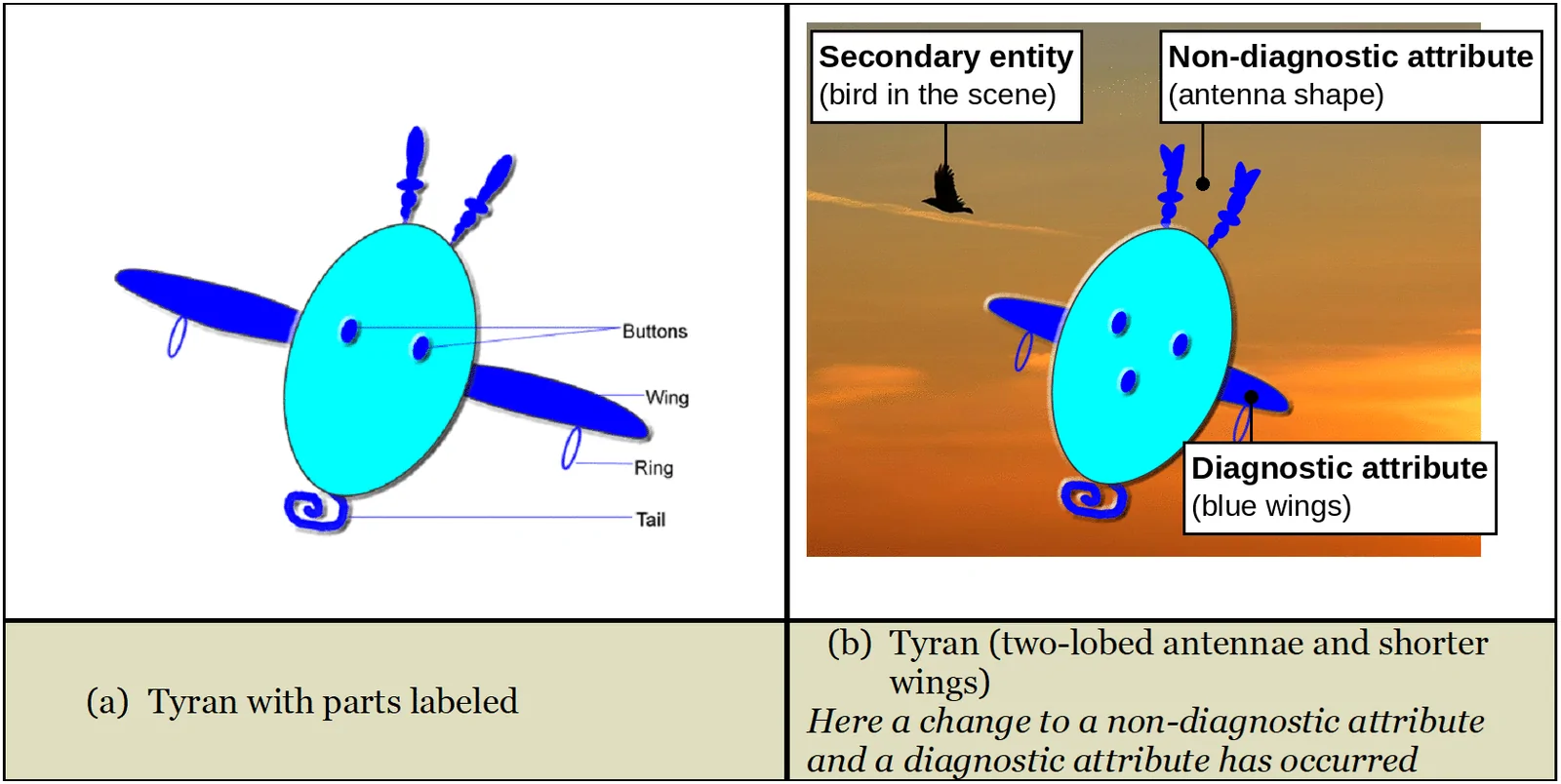
Observation of Tyran variants. **(a)** Shows standard Tyran with labeled parts. **(b)** Illustrates a variant with changes to a diagnostic (wing shape) and non-diagnostic (antenna type) attribute. 1(b) identifies a diagnostic attribute, a non-diagnostic attribute, and a secondary entity in a single scene.

### Eye tracking and derived measures.

Eye-tracking data provided objective measures of visual attention independent of conscious reporting decisions. Features that received no fixation had less opportunity for detailed evaluation, which distinguishes attentional allocation from reporting decisions.

Visual attention was recorded using a Tobii 4C eye-tracking system (60 Hz sampling). The Tobii SDK was integrated directly into the experimental application to record gaze behavior throughout each session. The eye tracker was positioned below a 24-inch LED display (1920 × 1080 pixels), and participants were seated at a viewing distance of approximately 60 cm. Fixations were defined as gaze points clustered within a 50-pixel radius lasting at least 200 milliseconds [31,32].

Dependent variables included:

- Fixation count: Number of fixations on diagnostic and on non-diagnostic attributes.
- Fixation duration: Total duration of fixations on diagnostic and on non-diagnostic attributes (milliseconds).

Saccade-to-fixation ratios were also computed for exploratory analyses of scanning patterns (S4 Appendix in S1 File).

### Verbal protocols.

Participants described each image aloud while viewing it. Audio recordings were transcribed and coded by two independent coders for the following hypothesis-relevant measures:

- **Diagnostic attribute variations:** Whether the participant mentioned a change to a category-defining feature between the training exemplars and the current image.
- **Non-diagnostic attribute variations:** Whether the participant mentioned a change to a category-irrelevant feature (e.g., antenna shape, orientation) between the training exemplars and the current image.
- **Secondary entity reporting:** This includes secondary entity mentions and secondary entity attribute descriptions.
- **Classification transference:** The difference between negative descriptors (references to missing trained-category features, e.g., “it doesn't have blue wings”) and positive descriptors (new attributes described on their own terms, e.g., “it has red spots”). Higher scores indicate greater reliance on trained schemas; lower scores indicate more flexible, attribute-based description. Computed only for catch items (5, 10, 15, 20).

Coding covered all verbal protocol observations (60 participants × 20 images). Agreement was assessed at the participant level using intraclass correlation coefficients (ICC(2,1), two-way random, single measures). Reliability was strong across all three hypothesis-relevant measures. Diagnostic variation reporting reached ICC = .87, non-diagnostic variation reporting exceeded  .99, and secondary entity reporting reached  .97 (S3 Appendix in S1 File). Both coders produced identical patterns of statistical significance at the group level. Non-diagnostic variation reporting differed significantly across groups under both codings (Coder 1: *F*(2, 57) = 5.53, *p* = .006; Coder 2: *F*(2, 59) = 5.91, *p* = .005); secondary entity reporting was significant under both coders (Coder 1: *F*(2, 57) = 6.43, *p* = .003; Coder 2: *F*(2, 59) = 4.75, *p* = .012); diagnostic variation reporting was non-significant under both coders. All analyses reported below use Coder 1's resolved coding.

### Blindness assessment procedures.

Change blindness was operationalized as the failure to explicitly report variations in diagnostic or non-diagnostic attributes when these altered features were fixated on.

Inattentional blindness was measured by embedding secondary entities, that is visually noticeable but task-irrelevant elements, in the scenes. Failure to report these entities after fixations was recorded as inattentional blindness.

Classification transference was assessed using four images (5, 10, 15, and 20). Objects in catch items had distinct colors and shapes, to test whether previously learned classifications were incorrectly applied (Fig 2).

**Fig 2:**
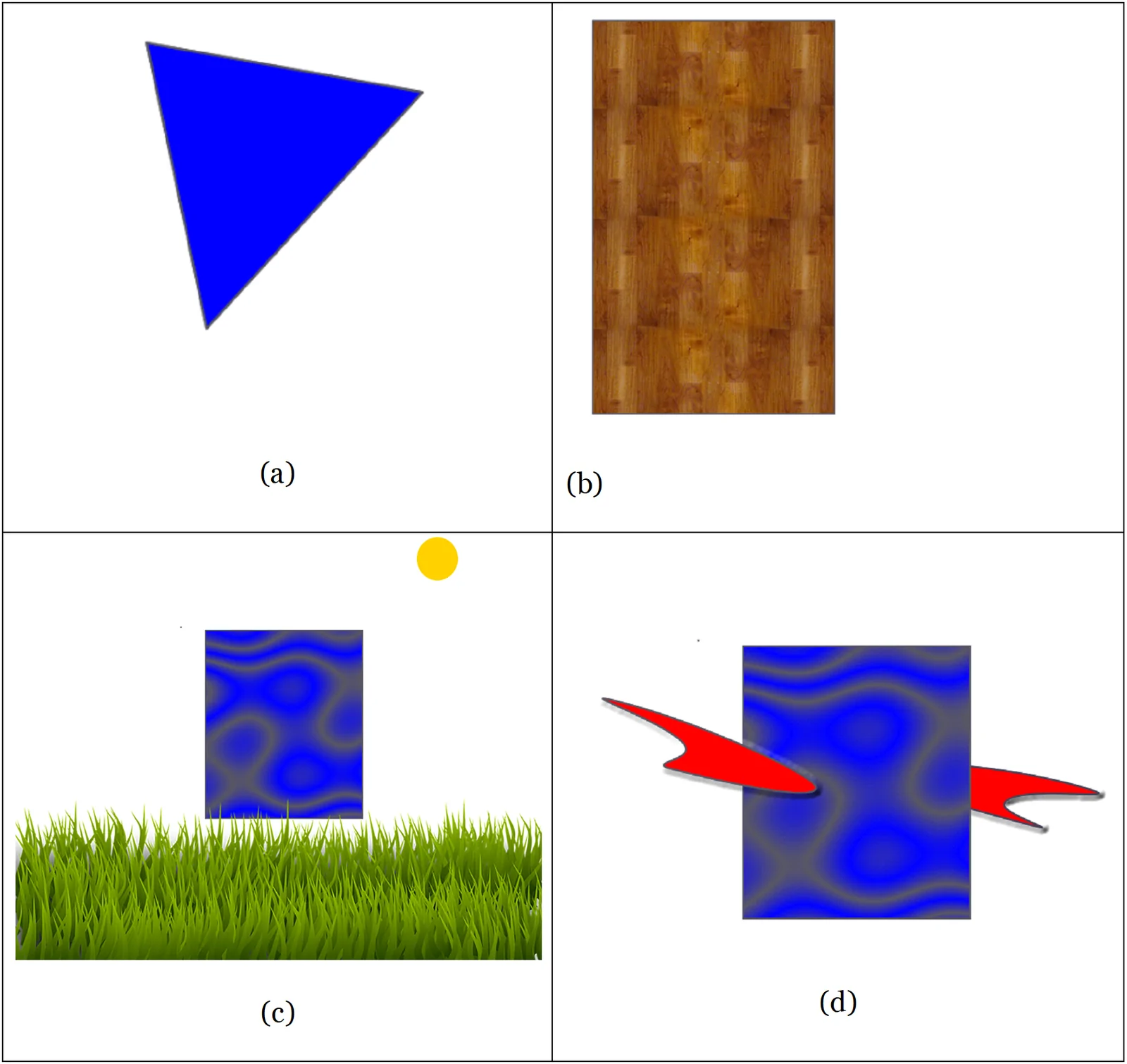
Catch items used to assess classification transference. **(a)** Image 5, **(b)** image 10, **(c)** image 15, **(d)** image 20. None shares diagnostic attributes with the trained category. Any reference to trained-category features in observers’ descriptions reflects expressive constraint and not perceptual similarity.

For each catch item, we coded whether observers (a) stated the entity was not a Tyran, (b) described it by listing diagnostic attributes it lacked (e.g., “it doesn't have blue wings”), or (c) described the entity's own attributes on its own terms (e.g., “it has brown patterns”). Responses (a) and (b) were counted as negative descriptors (ND); response (c) was counted as a positive descriptor (PD).

Classification transference scores were computed as ND − PD. Higher scores indicate greater reliance on trained schemas when describing novel entities. Fig 3 summarizes the experimental design and procedure.

**Fig 3:**
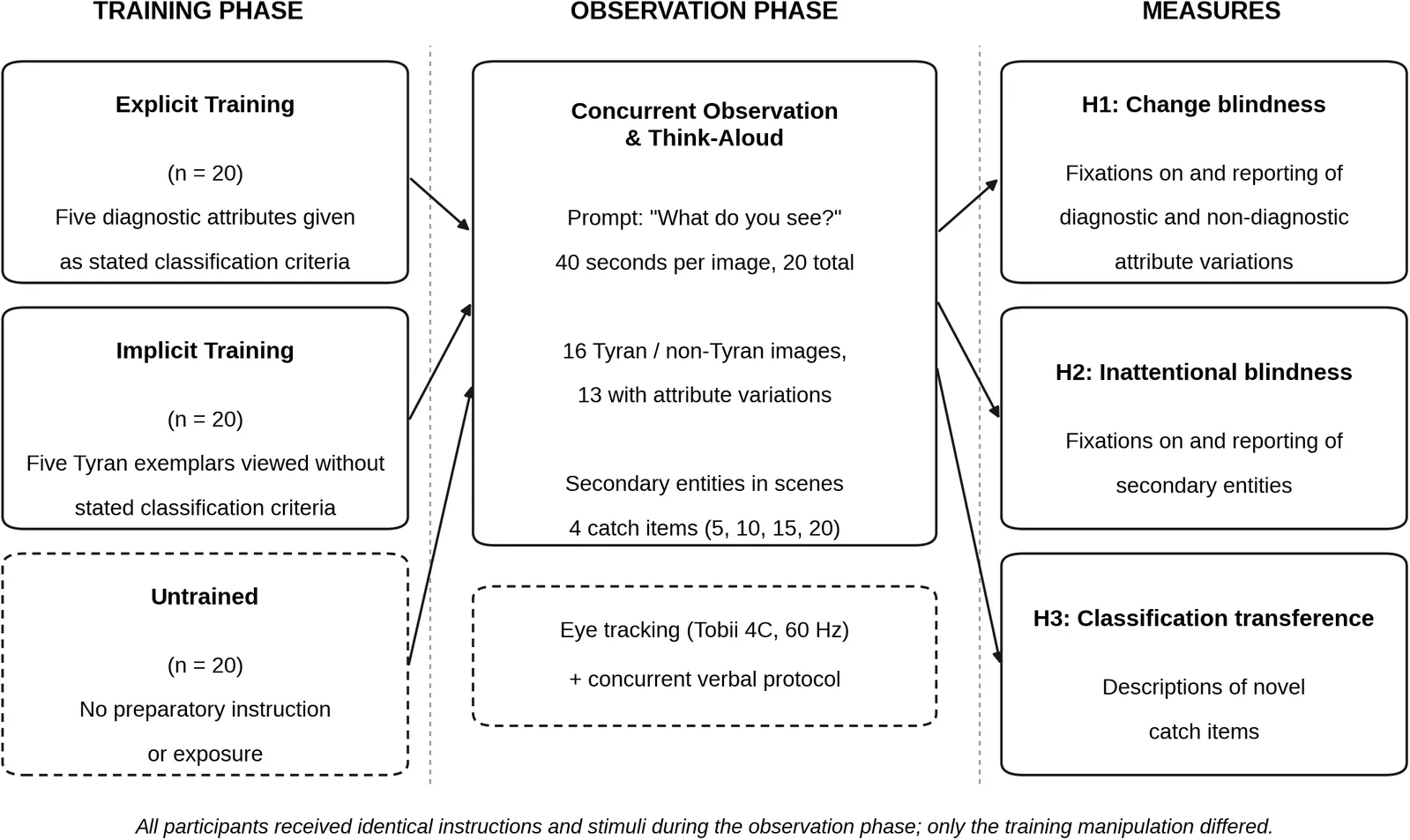
Experimental design and procedure.

Participants were randomly assigned to one of three training conditions: explicit training with stated classification criteria, implicit training with exemplars and no stated criteria, or no training. Each then completed a concurrent observation and think-aloud task with the same 20 images, presented in a fixed order.

### Participants and ethical compliance.

Data for the eye-tracking experiment were collected over a period of 11 days. Participants were undergraduate students aged 20–24, all in their third or fourth year of a Multimedia program, recruited through the department's social media channels. Forty-four participants were male and 13 were female; sex was not reported for three. None had prior experience with the classification task or the stimuli. Participation was voluntary, and participants received a small voucher for refreshments on completion of the session. All procedures were approved by the Interdisciplinary Committee on Ethics in Human Research at Memorial University of Newfoundland, Canada (ICEHR 20181757-BA) and, on that basis, by the ethics committee of Tabriz Islamic Art University, Iran, where the eye-tracking study was conducted. All participants gave informed consent. Before the session they were told what the study involved, including the eye-tracking equipment and the biometric data it records, which are gaze position, fixation patterns, and pupil dynamics. They were also told their verbalizations would be recorded.

The consent process explained how the data would be anonymized and used to study visual attention during classification tasks. Participants were informed of their right to withdraw from the study at any time without penalty and were given the option to request that their eye-tracking data be deleted after participation. All data were stored securely on encrypted servers, with identifying information kept separate from experimental data to protect participant confidentiality.

### Statistical analysis.

Each participant viewed twenty images, producing correlated observations within individuals. All Study 2 dependent variables were therefore analyzed using linear mixed models with training group (Explicit, Implicit, Untrained) as a fixed effect and participant as a random intercept [33,34]. Untrained is the reference level, so each coefficient gives the difference between a trained group and the untrained group in the dependent variable's own units. Pairwise comparisons use *z*-statistics from the model coefficients. Effect sizes are Cohen's d computed on participant means, consistent with the participant as the unit of randomization.

Models were fitted in Python using the statsmodels MixedLM procedure with restricted maximum likelihood estimation. The dependent variables were fixation count and total fixation duration for the eye-tracking hypotheses (H1a, H1c, H2a) and reporting rate for the verbal hypotheses (H1b, H1d, H2b). Classification transference (H3) was computed as the difference between negative and positive descriptors on catch images.

For eye-tracking and verbal analyses, images that did not contain the relevant attribute variation were excluded (see S2 Appendix in S1 File) because a variation that is absent cannot be fixated or reported. Nine images carried diagnostic variations and eight carried non-diagnostic variations, together accounting for the thirteen modified images described above. Catch images (5, 10, 15, 20) were excluded from all analyses except classification transference. After exclusion the analyses used 540 observations for diagnostic variations, 480 for non-diagnostic variations, 780 for secondary entities and 240 for classification transference.

## Results

We first examined whether training influenced visual attention patterns, as measured by eye-tracking, before analyzing verbal reporting behavior.

**H1: Training and attention to attribute variations**

**Non-diagnostic attribute fixations (H1a).** Explicitly trained participants fixated significantly less on non-diagnostic attributes than untrained participants in both count and duration (both *p* ≤  .007). Implicitly trained participants did not differ from untrained participants on either measure (Table 6). H1a is partially supported. Explicit training selectively reduced visual sampling of non-diagnostic attributes, while implicit training did not alter attention to these features (Fig 4A).

**Table 6:** Linear mixed models for eye-tracking measures (participant random intercept; reference = Untrained).

Measure	Explicit *M* (*SD*)	Implicit *M* (*SD*)	Untrained *M* (*SD*)	Explicit vs Untrained: β, *z*, *p*, *d*	Implicit vs Untrained: β, *z*, *p*, *d*
Non-diag. fix. count (H1a)	3.82 (4.21)	5.52 (5.17)	5.81 (5.16)	−1.99, −3.49, <.001, *d* = −1.37	−0.29, −0.50, .614, *d* = −0.14
Non-diag. fix. duration ms (H1a)	61.69 (141.52)	109.24 (129.91)	104.79 (104.95)	−43.10, −2.69, .007, *d* = −0.93	+4.45, +0.28, .781, *d* = +0.09
Diagnostic fix. count (H1c)	9.61 (6.83)	9.69 (7.40)	13.32 (8.05)	−3.71, −2.31, .021, *d* = −0.74	−3.63, −2.25, .024, *d* = −0.68
Diagnostic fix. duration ms (H1c)	176.17 (172.79)	173.13 (200.11)	254.80 (210.26)	−78.63, −2.15, .032, *d* = −0.68	−81.67, −2.23, .026, *d* = −0.64
Sec. entity fix. count (H2a)	7.95 (6.11)	8.72 (6.51)	10.06 (7.15)	−2.12, −2.45, .014, *d* = −0.74	−1.34, −1.56, .120, *d* = −0.47
Sec. entity fix. duration ms (H2a)	189.53 (210.08)	211.35 (197.39)	250.50 (229.90)	−60.97, −2.13, .033, *d* = −0.61	−39.14, −1.37, .171, *d* = −0.41

Note. *N* = 20 participants per group. Observation counts vary by analysis (see S2 Appendix in S1 File). β = unstandardized coefficient (difference from Untrained).

**Fig 4:**
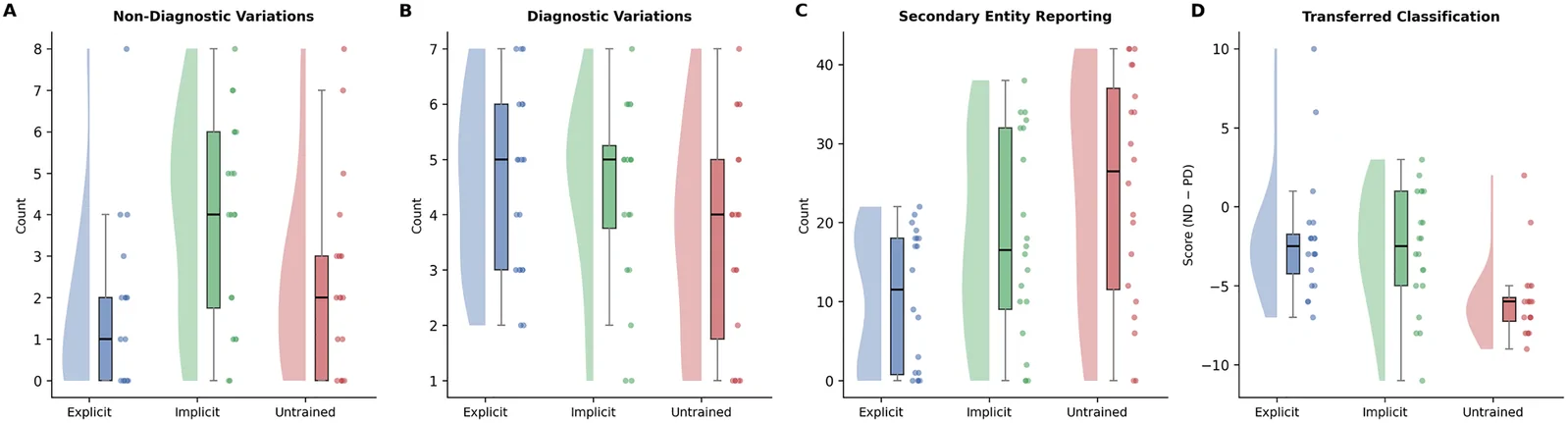
Fixation counts across training groups. Raincloud plots showing participant-level mean fixation counts. **(A)** Non-diagnostic attribute fixations (H1a): Explicitly trained participants showed significantly fewer fixations than untrained participants (*p* < .001); implicitly trained participants did not differ from untrained (*p* = .614). **(B)** Diagnostic attribute fixations (H1c): Both trained groups showed fewer fixations than untrained participants (both *p* ≤ .032). **(C)** Secondary entity fixations (H2a): Untrained participants fixated more than explicitly trained participants (*p* = .014); implicitly trained participants did not differ from untrained (*p* = .120).

**Non-diagnostic variation reporting (H1b).** Implicitly trained participants reported significantly more non-diagnostic variations than both untrained (*p* = .027) and explicitly trained participants (*p* = .001). Explicitly trained participants did not differ from untrained participants (Table 7). The ordering (Implicit > Untrained > Explicit) is consistent with H1b (Fig 5A).

**Table 7:** Linear mixed models for verbal reporting measures (participant random intercept; reference = Untrained).

Measure	Explicit *M* (*SD*)	Implicit *M* (*SD*)	Untrained *M* (*SD*)	Explicit vs Untrained: β, *z*, *p*, *d*	Implicit vs Untrained: β, *z*, *p*, *d*
Non-diagnostic variations (H1b)	0.19 (0.40)	0.49 (0.50)	0.29 (0.45)	−0.09, −1.04, .299, *d* = −0.34	+0.20, 2.22, .027, *d* = +0.67
Diagnostic variations (H1d)	0.50 (0.50)	0.49 (0.50)	0.39 (0.49)	+0.11, 1.85, .065, *d* = +0.58	+0.10, 1.66, .096, *d* = +0.51
Secondary entity reporting (H2b)	0.78 (0.92)	1.32 (1.67)	1.85 (1.81)	−1.06, −3.50, <.001, *d* = −1.13	−0.53, −1.74, .083, *d* = −0.49
Classification transference (H3)	−0.50 (1.44)	−0.66 (1.30)	−1.49 (1.29)	+0.99, 3.58, <.001, *d* = +1.18	+0.83, 2.99, .003, *d* = +1.03

Note. Per-image means and SDs. Images without the relevant attribute variation were excluded (S2 Appendix in S1 File). *N* = 20 participants per group. Observation counts: 540 diagnostic variations, 480 non-diagnostic variations, 780 secondary entities, 240 classification transference (catch items only).

**Fig 5:**
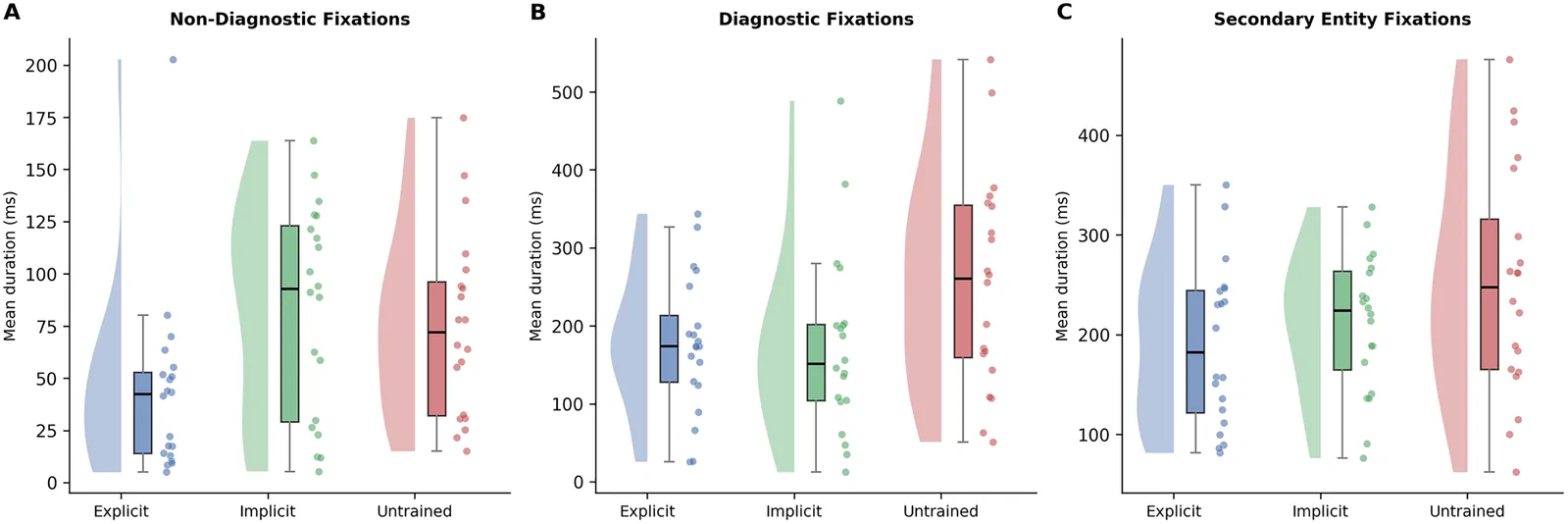
Verbal reporting measures across training groups. Raincloud plots show the distribution (half-violin), central tendency (box plot with median), and individual participant scores (*N* = 20 per group). **(A)** Non-diagnostic variation reporting (H1b): Implicitly trained participants reported significantly more non-diagnostic variations than both explicitly trained (*p* = .001) and untrained participants (*p* = .027). **(B)** Diagnostic variation reporting (H1d): No significant differences across groups. **(C)** Secondary entity reporting (H2b): Untrained participants reported significantly more secondary entity attributes than explicitly trained participants (*p* < .001). **(D)** Classification transference (H3): Both trained groups showed significantly higher classification transference scores than untrained participants (Explicit vs Untrained: *p* < .001; Implicit vs Untrained: *p* = .003).

**Diagnostic attribute fixations (H1c).** Untrained participants fixated significantly more on diagnostic attributes than both trained groups in count and duration (all *p* ≤ .032). H1c is supported. Trained participants processed diagnostic attributes more efficiently, requiring fewer and shorter fixations (Table 6, Fig 4B).

**Diagnostic variation reporting (H1d).** Reporting of diagnostic variations did not differ across groups (Table 7). Despite fixating less on diagnostic attributes, trained participants reported diagnostic changes at rates that did not differ from untrained participants. H1d is supported. Training did not impair diagnostic reporting (Fig 5B).

**H2: Training and attention to secondary entities**

**Secondary entity fixations (H2a).** Untrained participants fixated significantly more on secondary entities than explicitly trained participants (count: *p* = .014; duration: *p* = .033). Implicitly trained participants did not differ from untrained participants on either measure (count: *p* = .120; duration: *p* = .171). H2a is partially supported. Explicit training reduced peripheral visual exploration, while implicit training left it unchanged (Table 6, Fig 4C).

**Secondary entity reporting (H2b).** Untrained participants reported significantly more secondary entities than explicitly trained participants (*p* < .001). Implicitly trained participants fell between the two groups but did not differ significantly from either (Table 7). The ordering (Untrained > Implicit > Explicit) is consistent with H2b (Fig 5C).

**H3: Classification transference**

Both trained groups showed significantly higher classification transference scores than untrained participants, which shows greater reliance on trained-category references when describing novel entities (Table 7). The two trained groups did not differ from each other. H3 is supported (Fig 5D).

Table 6 presents the linear mixed model results for eye-tracking measures. Table 7 presents the linear mixed model results for verbal reporting measures. Figs 4 and 5 show raincloud plots displaying the distribution, central tendency, and individual data points for each measure.

Beyond the hypothesis-driven analyses, we examined whether training affected scanning behavior in regions outside the defined areas of interest (AOIs).

### Exploratory analysis: Attention to non-target regions.

To examine whether training reduced overall visual exploration or redirected it, we analyzed saccade-to-fixation count ratios (SC/FC) in regions containing no task-relevant features (non-AOI regions). Explicitly trained participants showed significantly higher SC/FC ratios in these regions (*M* = 1.43) than implicitly trained (*M* = 0.71; *p* < .001) and untrained participants (*M* = 0.65; *p* < .001). Implicitly trained and untrained participants did not differ (*p* = .781). SC/FC ratios within diagnostic, non-diagnostic, and secondary entity regions showed the opposite pattern, with explicitly trained participants exhibiting the lowest ratios. Fixation heatmaps illustrating these group-level attentional distributions are presented in S4 Appendix in S1 File.

### Robustness checks.

To test the robustness of the verbal reporting findings across a different population and response format, we conducted the same experiment separately with 84 participants (28 per group) at Memorial University of Newfoundland, Canada, using the same stimuli, training conditions, and prompt (“What do you see?”). Participants were undergraduate students (36 male, 48 female) who provided written responses on answer sheets instead of verbal ones. No eye-tracking was used. This study was covered by the same Memorial University ethics approval. Nine participants from an initial sample of 93 were excluded due to incomplete reports, inaccurate catch item responses, or illegibility, and group sizes were equalized to 28 by excluding the last-enrolled participants. Data were analyzed using the same linear mixed model specification as the main study.

The pattern of results was consistent with the main study for all three testable measures (Table 8). Diagnostic variation reporting did not differ across groups. Non-diagnostic variation reporting showed the same ordering, with implicitly trained participants reporting significantly more than both untrained and explicitly trained participants. Secondary entity reporting showed the same gradient, with untrained participants reporting more than both trained groups.

**Table 8:** Robustness check: linear mixed models for verbal reporting measures (*N* = 28 per group).

Measure	Explicit *M* (*SD*)	Implicit *M* (*SD*)	Untrained *M* (*SD*)	Explicit vs Untrained: β, *z*, *p*, *d*	Implicit vs Untrained: β, *z*, *p*, *d*
Non-diagnostic variations	0.005 (0.07)	0.11 (0.33)	0.03 (0.16)	−0.02, −0.92, .356, *d* = −0.47	+0.08, 3.51, <.001, *d* = +0.78
Diagnostic variations	0.04 (0.20)	0.04 (0.19)	0.02 (0.14)	+0.02, 1.21, .226, *d* = +0.34	+0.02, 0.97, .333, *d* = +0.28
Secondary entity reporting	1.10 (1.47)	1.85 (1.83)	2.71 (1.72)	−1.62, −4.94, <.001, *d* = −1.42	−0.86, −2.62, .009, *d* = −0.70

### Sensitivity to distributional assumptions.

No H1 or H2 conclusion depends on the distributional assumption. The eye-tracking and reporting measures are binary or count-valued, so we refitted each with a generalized estimating equation and cluster-robust standard errors by participant. Fixation and reporting counts were refitted as Poisson, fixation durations as negative binomial, and the binary reporting measures as logistic. Every contrast retained its direction and significance under the refit (Table 9). Classification transference is a difference score with negative group means, so it has no count or binary analogue and is not refitted here; it is tested in Table 7.

**Table 9:** Sensitivity of Study 2 results to distributional assumptions.

Measure	Model	Explicit vs Untrained: *p*	Implicit vs Untrained: *p*
Non-diag. fix. count (H1a)	Poisson	<.001	.650
Non-diag. fix. duration (H1a)	Neg. binomial	.007	.777
Diagnostic fix. count (H1c)	Poisson	.017	.031
Diagnostic fix. duration (H1c)	Neg. binomial	.021	.040
Sec. entity fix. count (H2a)	Poisson	.015	.120
Sec. entity fix. duration (H2a)	Neg. binomial	.041	.167
Non-diag. variation reporting (H1b)	Logistic	.271	.036
Diagnostic variation reporting (H1d)	Logistic	.065	.104
Secondary entity reporting (H2b)	Poisson	<.001	.115

Note. *p*-values from generalized estimating equations with an exchangeable working correlation and cluster-robust standard errors by participant. Linear mixed model values for the same contrasts appear in Tables 6 and 7.

## Discussion

Entities, features, and behavioral patterns that are never fixated cannot be reported, and features that are never reported cannot lead to discovery. This paper demonstrates that training in citizen science constrains which features receive visual attention, establishing a perceptual boundary on discovery potential. Study 1 revealed consistent associations between training type and discovery outcomes across 206 confirmed discoveries. Study 2 provided experimental evidence identifying the cognitive processes behind these patterns. Training narrows visual sampling, reduces attention to secondary entities, and constrains how observers describe novel entities.

Explicitly trained observers predominantly reported targeted discoveries aligned with project goals, while untrained observers accounted for the majority of opportunistic and serendipitous discoveries. These patterns suggest that training is associated with greater focus on diagnostic attributes and reduced sensitivity to unexpected elements. While these observational data cannot establish causation, given that training type and platform are aligned in this dataset, they motivated our controlled experimental investigation.

Study 2 tested these observations under controlled conditions using eye-tracking and verbal reports. Explicitly trained participants showed attentional narrowing. They fixated less on non-diagnostic attributes and reported fewer non-diagnostic variations, consistent with change blindness for category-irrelevant features. Diagnostic variation reporting, however, was unaffected by training. The invariance carries weight only alongside the fixation data. Trained observers fixated diagnostic attributes less often and for shorter durations, yet reported diagnostic changes at the same rate as untrained observers. Non-diagnostic attributes show the opposite pairing. Fewer fixations there produced fewer reports. Training therefore redistributed attention across feature types while preserving the reporting of features it prioritized [35,36]. Explicit training also left participants seldom reporting prominent secondary entities, even ones irrelevant to the task. Untrained observers explored more broadly. Implicitly trained participants showed the highest rates of non-diagnostic variation reporting, exceeding both explicitly trained and untrained groups. For secondary entity reporting and fixation measures, implicitly trained participants did not differ significantly from untrained participants.

The phenomenon of classification transference explains reduced detection of out-of-scope entities. Faced with such entities, explicitly trained participants described them using familiar diagnostic attributes, leaving the novelty unreported. This effect, separate from simple misclassification [29], reflects learned schemas that constrain how observers frame new information [28].

These findings reveal a structure to the training-discovery paradox that is more severe than attentional narrowing alone. Discovery requires passing through two independent gates. The observer must first fixate on an entity or a feature, and then must be able to describe it without defaulting to trained categories. Training closes both. Gate one is perceptual. Trained observers do not fixate on non-diagnostic attributes and secondary entities. This constrains what is available to report. Gate two is expressive. Even when trained observers encounter entities they recognize as novel, they describe them by referencing what is missing from trained categories instead of reporting what is present. Either gate alone is sufficient to prevent discovery. Training closes both simultaneously.

The attentional data reveal a further pattern that efficiency alone does not explain. Training preserved the total volume of visual exploration while redirecting it away from potentially informative features toward uninformative space. Fixation counts on diagnostic attributes decreased, consistent with efficient processing, but scanning activity in regions containing no task-relevant features increased. Explicitly trained participants showed saccade-to-fixation ratios in empty regions more than twice those of untrained observers. The trained observer appears efficient. The platform receives accurate classifications. Neither party has visibility into what is being missed.

This redistribution of visual exploration has a specific consequence for implicit training. Implicitly trained observers did not show the attentional misdirection exhibited by the explicitly trained group. They sampled as broadly as untrained observers while reporting more non-diagnostic variations than either group. Implicit training may therefore represent a distinct perceptual orientation, one that preserves attentional breadth while sharpening sensitivity to variation. If this finding generalizes, it suggests that the training-discovery tradeoff is not inevitable. The question is not whether to train, but how.

The inability to separate “true” perceptual constraints from reporting priorities reflects the ecological reality of citizen science. When participants do not report unexpected features, the distinction between “didn't see” and “didn't think it mattered” may be false. Training makes certain features psychologically invisible by defining them as irrelevant before conscious evaluation occurs. Despite identical instructions across all conditions asking simply “What do you see?,” training shaped how observers interpreted this neutral prompt. This demonstrates that training does more than add knowledge. It creates perceptual and evaluative frameworks that can override subsequent instructions. The power of training to shape observation persists even when observers are given completely open-ended prompts, which shows how these frameworks become embedded.

Fig 6 summarizes the relationships between training, cognitive constraints, and discovery outcomes.

**Fig 6:**
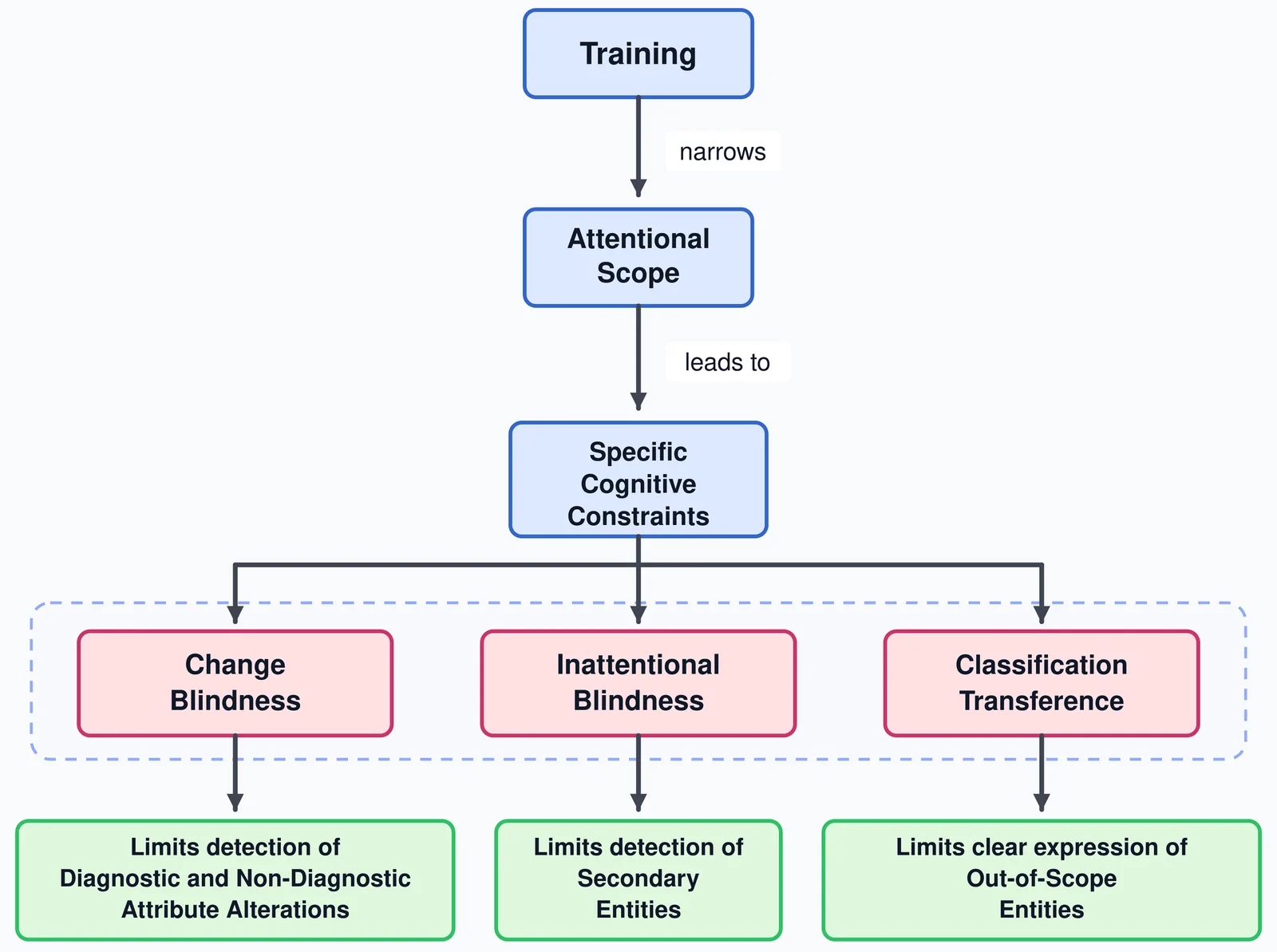
Conceptual model illustrating how explicit training-induced cognitive constraints (change blindness, classification transference, inattentional blindness) constrain visual attention and reporting, and so constrain discovery outcomes.

Our findings suggest that platforms seeking both classification accuracy and discovery potential could benefit from combining trained and untrained observation within the same workflow, or incorporating periods of open-ended exploration to counteract attentional narrowing. The specific design of such approaches would need to account for platform context and contributor dynamics, and their effectiveness remains to be tested in active citizen science projects. The primary reason for training is classification accuracy. Adding contributors does not offset its cost. Citizen science gains reliability by pooling many contributions, but pooling corrects only errors that vary across observers. When common training directs contributors past the same feature, adding observers raises confidence in the recorded classifications without widening what was observed. Preliminary analyses using machine learning methods on the attribute data (S7 Appendix in S1 File) suggest that classification accuracy can be improved post hoc for all training conditions when observers report attributes in sufficient detail. This has a specific implication for the training-discovery tradeoff. The accuracy advantage of explicit training is recoverable through computational correction, but the attribute reporting that explicit training suppresses is not. Observers who report more attributes provide richer input for both discovery and automated refinement [37]. This asymmetry favors training approaches that maximize attribute reporting, even at the cost of initial classification accuracy, because accuracy can be recovered while unreported observations cannot. A non-diagnostic attribute is irrelevant to the current classification rule, not to a later scientific question. Its value cannot be known at collection, so attribute-level reports preserve options that a label alone does not.

Table 10 maps the practical consequences of training across conditions.

**Table 10:** Discovery tradeoff matrix: Effects of training on discovery outcomes.

Dimension	Explicit Training	Implicit Training	Untrained
Perceptual Scope	Narrow, diagnostic-focused	Broad, with heightened sensitivity to variation	Broad, undirected
Cognitive Constraint	Change blindness (non-diagnostic) + classification transference	Moderate filtering	Low filtering
Anomaly Sensitivity	Low	Moderate to high	High
Discovery Type Enabled (Study 1)	Targeted	Targeted + serendipitous	Opportunistic and serendipitous
Suggested system role	Classification and verification	Hypothesis refinement	Exploration and anomaly detection

Note. This matrix summarizes the pattern of results in interpretive terms. The characterizations are qualitative judgments drawn from the quantitative results in Tables 6–8, not additional measurements.

Both studies converge on the same account across naturalistic and laboratory contexts. This combination of archival and experimental evidence is uncommon in citizen science research, where studies rely on one approach or the other. The systematic patterns observed in actual citizen science platforms (Study 1) align with the specific cognitive mechanisms identified in our controlled experiments. Untrained contributors in biodiversity platforms made proportionally more opportunistic discoveries, consistent with our experimental findings of reduced inattentional blindness among untrained observers, who had no trained category to impose on novel entities. Explicitly trained Zooniverse participants primarily produced targeted discoveries, mirroring the attentional narrowing we quantified through eye-tracking. Neither approach alone provides sufficient evidence of the causal role of training in discovery outcomes; together, they reveal consistent patterns across diverse contexts and measurement approaches. The robustness check confirmed these verbal reporting patterns with a different population and written responses, further supporting their generalizability.

The opening case fits this account. Hanny van Arkel had no training in galaxy morphology, and the object she reported had passed through the attention of observers who did. Trained observers were less likely to fixate the anomaly, and less able to describe it except as a departure from known morphologies. Preserving discovery potential requires maintaining perceptual flexibility. Training should improve classification accuracy while safeguarding the observer's ability to perceive phenomena beyond existing knowledge frameworks [38,39].

## Limitations

Our findings must be interpreted in light of several limitations.

First, our experimental design cannot definitively separate perceptual, attentional, and reporting mechanisms. We cannot determine whether fixated but unreported features reflect perceptual filtering or conscious reporting decisions. However, the eye-tracking data constrain the inference in one direction. A feature that received no fixation was unlikely to be evaluated in detail and reported, while a fixated feature may or may not be reported [40]. The consistent absence of fixations on unreported features, combined with identical instructions across all conditions, suggests that training shaped visual attention itself and not only reporting decisions.

Second, we cannot assess the scientific value of different observation types. In Study 1, we analyzed only confirmed discoveries, but cannot determine how many irrelevant observations untrained observers might generate alongside these discoveries. That ratio of signal to noise remains unmeasured and matters for platforms where efficiency is valued alongside discovery potential.

Third, our use of artificial stimuli eliminates specific prior knowledge about Tyrans but cannot control for domain-general observation skills. Random assignment should distribute these capabilities across conditions, but individual differences in baseline observation skills might moderate training effects. Artificial stimuli also may not fully represent the complexity and contextual richness of real scientific observations.

Fourth, Study 1's implicit training category contained only 10 discoveries, and while permutation tests confirmed this comparison (*p* = .029; S1 Appendix in S1 File), the small sample warrants caution. Study 2's sample of 60 participants (20 per group) is modest, though the robustness check with 84 additional participants reproduced the verbal reporting findings.

Fifth, our study captures observation within a single sitting. While most citizen science contributions involve single or brief observations, some contributors participate repeatedly over extended periods, during which training effects may evolve.

Finally, some citizen science platforms employ hybrid training approaches, and contributors often self-select into projects based on interest and prior expertise. Platform-specific factors such as interface design, community norms, and feedback mechanisms may interact with training in ways our design cannot capture.

These limitations point toward several open questions. Can hybrid training balance accuracy with discovery potential? How do individual differences in cognitive style interact with training effects? Addressing these will require longitudinal studies and field experiments in active citizen science projects.

## Conclusion

Training improves classification accuracy by directing attention to the features a category defines. It reduces attention to everything else. Across 206 confirmed discoveries and two experiments, contributors without structured training accounted for most discoveries falling outside project scope, and explicitly trained observers fixated 34% less on non-diagnostic features. Classification transference constrains how a novel entity can be described even once it is noticed.

A missed variation or anomaly appears in neither the correct nor the incorrect column of any accuracy measure, and pooling more observers does not recover it, because common training carries every observer past the same feature. Accuracy can improve while the range of phenomena a system records narrows. Nothing in the mechanism is specific to citizen science; the same constraint should hold wherever standardized training meets variable phenomena, in medical diagnosis, security screening, and quality inspection. Citizen science platforms and other systems that depend on trained observation therefore need to assess what their observers are prepared to notice, not only how accurately they classify.

## Supporting information

S1 Fig Aggregated fixation-density heatmaps across training conditions. Heatmaps show the aggregated distribution of fixations for explicitly trained (EG), implicitly trained (IG), and untrained (UG) participants. Fixations in the explicitly trained condition were more concentrated around task-relevant regions than those in the other conditions. All panels use identical color-map scaling to permit direct comparison of fixation density across conditions.
(TIF)

S1 File Supporting appendices S1–S7. This file contains the supplementary methods, analyses, tables, and figures reported in Appendices S1–S7.
(DOCX)
