# Supplementary material for: The training–discovery paradox: Evidence from citizen science
Source: PLoS One. 2026 Oct 29;21(10):e0361148. doi: 10.1371/journal.pone.0361148 (PMC13719020; doi:10.1371/journal.pone.0361148)
Supplement: Supplementary file 1 [file pone.0361148.s002.docx]

**S1 Appendix. Robustness Analyses for Study 1**

**Permutation Tests**

To assess the robustness of the association between training type and discovery type, we conducted permutation tests using chi-square as the test statistic (100,000 permutations; seed = 42). For the overall 3×3 association, group labels were shuffled while preserving marginal totals. Pairwise comparisons used the same procedure on the relevant 2×3 subtables. We also conducted Fisher's exact tests on 2×2 tables collapsing discovery type into targeted versus non-targeted.

**Table A1.** Permutation and Fisher's exact tests for Study 1 discovery-type comparisons.

| Comparison | Permutation χ² | Permutation p | Fisher's exact p (2×2) |
| --- | --- | --- | --- |
| Overall (3×3) | 41.29 | < .001 | — |
| Explicit vs Untrained | 39.25 | < .001 | < .001 |
| Implicit vs Untrained | 7.16 | .029 | .013 |
| Explicit vs Implicit | 2.63 | .222 | 1.00 |

*Note.* Permutation tests: 100,000 permutations. Fisher's exact tests collapsed discovery types into targeted vs non-targeted. The overall test used the full 3×3 table; pairwise tests used 2×3 subtables.

All three pairwise comparisons produced results consistent with the primary analysis. The Implicit vs Untrained comparison, which is constrained by the small Implicit group (n = 10), reached significance under both the permutation test and Fisher's exact test.

**S2 Appendix. Image Exclusion Details for Study 2**

**Table A2.** Image exclusions per eye-tracking analysis.

| **Analysis** | **Excluded images** | **Analyzed images** | **Obs per group** | **Total** |
| --- | --- | --- | --- | --- |
| Diagnostic fixations | 1, 5, 6, 8, 9, 10, 11, 14, 15, 19, 20 | 2, 3, 4, 7, 12, 13, 16, 17, 18 | 180 | 540 |
| Non-diagnostic fixations | 2, 3, 5, 6, 8, 10, 11, 12, 15, 17, 18, 20 | 1, 4, 7, 9, 13, 14, 16, 19 | 160 | 480 |
| Secondary entity fixations | 5, 6, 10, 14, 15, 18, 20 | 1, 2, 3, 4, 7, 8, 9, 11, 12, 13, 16, 17, 19 | 260 | 780 |

Each stimulus image contained a specific combination of attribute types. Catch images (5, 10, 15, 20) were excluded from all eye-tracking analyses as they were designed to assess classification transference. Additional images were excluded when they did not contain the relevant attribute type. A single exclusion set applies to both non-diagnostic measures, since an image either carries a non-diagnostic variation or it does not.

No images were excluded from the verbal reporting analyses. Classification transference was analyzed only on catch images.

**S3 Appendix. Inter-Rater Reliability for Verbal Protocol Coding**

Two independent coders scored all 1,200 verbal protocol observations (60 participants × 20 images). Agreement was assessed at the participant level using intraclass correlation coefficients (ICC(2,1), two-way random, single measures).

**Table A3.** Inter-rater reliability for verbal coding measures.

| **Measure** | **ICC(2,1)** |
| --- | --- |
| Diagnostic variation reporting (H1d) | .87 |
| Non-diagnostic variation reporting (H1b) | > .99 |
| Secondary entity reporting, SE+SEA (H2b) | .97 |

*Note.* ICCs computed on participant-level totals (N = 60). All analyses use Coder 1's resolved coding.

**S4 Appendix. Saccade-to-Fixation Count Ratios and Fixation Heatmaps**

Saccade-to-fixation count ratios (SC/FC) quantify exploratory scanning behavior within each region of interest [40]. Higher ratios indicate greater exploratory scanning; lower ratios indicate focused attention.

SC/FC ratios within diagnostic attribute regions did not differ across training conditions (Explicit: M = 0.46; Implicit: M = 0.60; Untrained: M = 0.76; all pairwise p > .05). In non-diagnostic attribute regions, explicitly trained participants showed lower ratios (M = 0.41) than implicitly trained (M = 0.65; p < .001) and untrained participants (M = 0.72; p < .001). For secondary entities, explicitly trained participants showed the lowest ratios (M = 0.34) compared to untrained (M = 0.78; p < .001) and implicitly trained participants (M = 0.56; p = .004).

In non-AOI regions (areas containing no task-relevant features), explicitly trained participants showed higher SC/FC ratios (M = 1.43) than implicitly trained (M = 0.71; p < .001) and untrained participants (M = 0.65; p < .001). Implicitly trained and untrained participants did not differ (p = .781).

Fixation heatmaps were generated using OGAMA. S1 Fig illustrates group-level attentional distributions for Image 12.

**S1 Fig.** Aggregated fixation-density heatmaps across training conditions. Heatmaps show the aggregated distribution of fixations for explicitly trained (EG), implicitly trained (IG), and untrained (UG) participants. Fixations in the explicitly trained condition were more concentrated around task-relevant regions than those in the other conditions. All panels use identical color-map scaling to permit direct comparison of fixation density across conditions.

**S5 Appendix. Data and Code Availability**

All data and analysis code are available at https://doi.org/10.5281/zenodo.22240008.

**Study 1 data:** Discovery classifications with training type, discovery type, and platform. Platform confirmation records.

**Study 2 data:** Verbal protocol coding (primary coder, used in all analyses). Verbal protocol coding (second coder, for inter-rater reliability). Eye-tracking fixation data for diagnostic attributes, non-diagnostic attributes, and secondary entities.

**Analysis code:** Python script reproducing all Study 2 analyses, including effect sizes and the sensitivity analysis (requires Python 3.8+ with pandas, numpy, scipy, statsmodels, and openpyxl).

**S6 Appendix. Pretest**

**Pretest 1: Prompt Selection and Training Validation**
Twelve ecology students with field observation experience participated in the first pretest. We compared three prompts to identify which would elicit the fullest and least biased responses: (1) "Identify the entity," (2) "Report your sighting," and (3) "What do you see?" The first two prompts, though commonly used in classification tasks, constrained the responses they elicited. Trained participants given these prompts reported entity classification, ignored secondary entities, and omitted attribute descriptions. In contrast, "What do you see?", a variation of prompts used by established platforms like eBird, elicited fuller attribute descriptions and secondary entity reports across all training conditions. This prompt elicited descriptions unconstrained by classification while remaining close to the wording used on active platforms.

This pretest also validated our training materials. We compared two-attribute versus five-attribute classification rules to ensure implicit learners could infer patterns from examples and explicit learners could master the rules. While both rule sets yielded equivalent classification accuracy, participants reported the two-attribute version as insufficiently engaging, potentially affecting sustained attention. We therefore adopted the five-attribute rule for the main experiment.

Ecology students were selected for this pretest because their field observation experience approximates the domain familiarity of active citizen science contributors.

**Pretest 2: Timing Optimization**
Fifteen business students participated in a second pretest confirming our modifications. This pretest established 40 seconds as an adequate viewing duration, sufficient for full observation without inducing fatigue or rushed reporting, and consistent with prior work in citizen science on species identification [30]. Shorter durations (20-30 seconds) produced incomplete reports. Longer durations (50-60 seconds) produced no further attributes or entities. Business students with no ecology background were selected to approximate naive citizen science participants encountering an unfamiliar classification task.

Establishing that "What do you see?" produced the least biased responses across training conditions allows reporting differences to be attributed to training and not to the prompt. This matters given the ongoing debate about whether training effects reflect perceptual, attentional, or reporting mechanisms.

**S7 Appendix. Post Hoc Classification Accuracy**

To assess whether classification accuracy is recoverable from attribute-rich verbal reports, we compared raw classification accuracy across training conditions and tested whether machine learning methods could achieve comparable accuracy from untrained observers' attribute descriptions.

**Raw Classification Accuracy**

Explicitly and implicitly trained participants classified each entity as Tyran or non-Tyran during the observation task. We computed accuracy by comparing their classifications against ground truth. Explicitly trained participants achieved 86.2% overall accuracy (386/448 correct). Implicitly trained participants achieved 63.8% (286/448 correct).

**Table A4.** Classification accuracy by training condition.

| **Group** | **True Positive** | **False Positive** | **True Negative** | **False Negative** | **Overall Accuracy** |
| --- | --- | --- | --- | --- | --- |
| Explicit | 180 | 46 | 206 | 16 | 86.2% |
| Implicit | 132 | 98 | 154 | 64 | 63.8% |

**Machine Learning Classification of Untrained Reports**

Untrained participants did not classify entities into trained categories, so their accuracy cannot be computed directly. Instead, we tested whether the attributes they reported contained sufficient information for automated classification. We labeled the attribute data and applied two classifiers: Support Vector Machine (SVM) and Random Forest (RF), using 5-fold cross-validation to guard against overfitting.

**Table A5.** Classification accuracy from untrained attribute reports (5-fold cross-validation).

| **Fold** | **1** | **2** | **3** | **4** | **5** | **Mean** |
| --- | --- | --- | --- | --- | --- | --- |
| SVM | 0.679 | 0.889 | 0.778 | 0.815 | 0.815 | 79.6% |
| RF | 0.714 | 0.815 | 0.889 | 0.889 | 0.815 | 82.4% |

Untrained observers' attribute descriptions, when processed through standard classifiers, achieved 79.6-82.4% accuracy. This approaches the explicitly trained group's raw accuracy (86.2%) and exceeds the implicitly trained group's raw accuracy (63.8%).

**Attribute Completeness**

To test whether the richness of diagnostic attribute reporting differed across groups, we compared the mean number of distinct diagnostic attributes mentioned per entity. No significant differences were found (all p > .38), indicating that training did not increase the number of diagnostic attributes observers reported.

**Table A6.** Attribute completeness by training condition (Tukey HSD comparisons).

| **Comparison** | **Mean Diff** | **p** |
| --- | --- | --- |
| Explicit vs Implicit | +0.045 | .900 |
| Explicit vs Untrained | +0.145 | .383 |
| Implicit vs Untrained | +0.101 | .619 |

These results indicate that the accuracy advantage of explicit training is recoverable through computational methods applied to attribute-rich reports, while the attribute reporting that explicit training suppresses (as shown in the main analyses) is not recoverable.
